# Influence of Maternal Fish Intake on the Anthropometric Indices of Children in the Western Amazon

**DOI:** 10.3390/nu10091146

**Published:** 2018-08-23

**Authors:** Mônica P. L. Cunha, Rejane C. Marques, José G. Dórea

**Affiliations:** 1 Universidade Federal de Rondônia, Porto Velho 76801-059, Brazil; 2 Department of Nutrition, Faculty of Health Sciences, Universidade de Brasília, Brasília 70919-970, Brazil; jg.dorea@gmail.com; 3 Universidade Federal do Rio de Janeiro, Macaé 27930-560, Brazil; rejanecmarques@globo.com

**Keywords:** fish consumption, maternal, anthropometric indices, mercury

## Abstract

We studied trends in fish intake among pregnant women living in the Madeira River Basin in Rondônia State, Brazil, to investigate the influence of maternal fish intake on anthropometric indices of children followed up to 5 years. Maternal fish intake was assessed using hair mercury concentrations of mothers and children at delivery and 6, 24, and 59 months. Data analysis was performed using a linear mixed-effect model. Mothers were predominantly young, had low incomes and limited schooling, and breastfed for >6 months. Only 1.9% of children had low birth weight. Anthropometric indices in approximately 80% of the study population showed Z-score values ranging from ≥−2 to ≤1. The influence of maternal fish intake on anthropometric indices, including height-to-age (H/A), weight-to-age (W/A), and weight-to-height (W/H) were not statistically significant after model adjustments. However, higher income and larger birth weight had a positive influence on H/A and W/A, whereas W/H gain was favored by higher maternal educational status and breastfeeding duration. Other variables (hemoglobin concentration and maternal age) had a positive significant influence on anthropometric indices. Maternal fish intake (or its attendant MeHg exposure) did not affect children growth. Nevertheless, it is advisable to avoid mercury-contaminated fish during pregnancy and childhood.

## 1. Introduction

The promotion of adequate child growth is considered a global priority, especially in vulnerable populations. Evidence suggests that from conception to early life, good nutrition and a healthy environment can influence optimal growth, as well as ensure gains in human capital and long-term health [1]. Therefore, early life is a critical phase of human development that is particularly sensitive to environmentally caused pathologies and nutritional stimuli that may influence health outcomes later in life [2].

The impact of the environment and maternal nutrition on child growth and development has been previously studied. Specifically, maternal fish intake during pregnancy is known for its benefit in neurodevelopment [3], intrauterine growth [4], birth weight, and a diminished risk of premature birth [5]. However, maternal fish consumption may also expose the developing fetus to environmental pollutants [6], which may disturb the endocrine system and thereby affect weight gain [7].

It is believed that fish intake during pregnancy can influence not only the developing fetus, but also later child growth. A large, multicenter study that analyzed data from 26,184 pregnant women, as well as their respective offspring, reported that fish consumption during pregnancy (>3 times per week) was associated with rapid growth and increased adiposity in European and American children [2]. Since several factors may influence food consumption and lifestyle in different populations, generalization of these findings should be done with care.

Fish is recognized as an important source of vitamins, iodine, long-chain polyunsaturated fatty acids; it also provides essential elements such as manganese (Mn), copper (Cu), and selenium (Se) [8,9] known to neutralize the toxic effects triggered by Hg [10]. People living in the Amazon region have plentiful access to rivers and consequentially consume fish as an important source of protein to complement staple starchy foods [11]. Under these circumstances, fish consumption is also associated with exposure to methylmercury (MeHg) [12,13]. The socio-demographic characteristics and habitual fish consumption among Amazonian mothers also exposes their children (during pregnancy and lactation) to MeHg. Indeed, in Amazonian populations, hair mercury (HHg) concentration has been established as an important marker for both the consumption of fish and MeHg exposure [13,14,15,16].

The intense urbanization processes that have occurred in the Amazon region have led to lifestyle changes and, consequently, changes in the diet of this population through the introduction of industrialized foods. However, fish remains an important dietary item, especially in traditional populations [14,17]. Currently, Amazonian children are a heterogeneous population with different lifestyles. Nevertheless, exposure to MeHg (from fish consumption), coupled with the lack of adequate infrastructure (roads, clean water, and waste disposal) and substandard living conditions, are still some of the major problems. A recent study has shown that unfavorable socio-economic conditions associated with poor feeding and nutritional practices in the Northern region of Brazil are still determinants of poor anthropometric indices among young children [18].

Given that both the environment and eating habits of mothers may determine the health of their children throughout life, this study aimed to investigate the influence of maternal fish consumption on the anthropometric indices height-to-age (H/A), weight-to-age (W/A), and weight-to-height (W/H) of children followed-up since birth until the age of 5 in the Western Amazon, Rondônia State, Brazil.

## 2. Materials and Methods

### 2.1. Study Population

We used data from a cohort study initiated in 2007 to evaluate the development of children exposed to environmental mercury. This study protocol (001/07 and 012/08) was approved by the Ethics Committee of the Federal University of Rondônia in 12 February 2006. Mothers or legal guardians of all participants provided written informed consent. The details of the sampling procedure have been described [14,15]. Briefly, the study participants consisted of pregnant women and their newborns living in the area nearby the Jamari, Madeira, and Mamoré rivers, according to the following criteria: women with healthy pregnancies, the absence of congenital malformations, and residing for at least 5 years in the study area. 

This was formed as an extension of previous cross-sectional work in the area [14,15] and extended to former riverines now living in urban areas, and nonurban families. The study started in 2006 when pregnant women were contacted. Information on the delivery condition (home or hospital), gestational age, and birth weight was obtained from hospital records or from midwives.

A total of 1668 pregnant women were invited to participate in this study, of which 215 refused to participate and 11 pregnancies ended in fetal death. Of the 1442 live births, 9 were excluded due to congenital abnormalities. In total, 1433 children were followed-up to the age of 5 years. Moreover, an earlier publication addressed fish consumption and birth weight. In all groups of mothers there was a highly significant correlation between fish consumption and total HHg concentrations: riverine (Spearman r = 0.8089, *p* = 0.001), urban (Spearman r = 0.7681, *p* = 0.001), rural (Spearman r = 0.7644, *p* = 0.001), and tin miner (Spearman r = 0.1211, *p* = 0.001); thus, confirming total HHg as a reliable biomarker of fish consumption [16].

The research team that conducted the interviews received specific training on how to administer the questionnaire to the mothers during visits, as well as how to weigh and measure the study children. The questionnaire has been used in our previous study [14,15] to collect information on fish intake, breastfeeding practices, and pertinent socioeconomic data. The periods (at birth, 6, 24 and 59 months) were selected because they understood phases that make up some growth and development milestone. Data regarding age, date of birth, and birth weight were obtained from hospital or midwife records. Through planned home visits, trained researchers performed interviews, anthropometric measurements, collected hair samples from both mothers and children, and screened anemic children (using hemoglobin tests); when appropriate, mothers were advised to seek available health and medical services. The researchers who collected the growth data were blind to the Hg levels of the subjects, and laboratory personnel responsible for HHg analyses were blinded to the growth and development data.

### 2.2. Anthropometric Data

The weight and length of newborns who were delivered in regional public hospitals were registered by the staff nurses. For mothers who gave birth at home, midwives, who had been previously identified by the residents, were trained by the project team to obtain the necessary information at birth. Weight and length at birth were measured with a portable infant scale that had a 1 g accuracy and a portable horizontal anthropometer (Seca, Hamburg, Germany), respectively. For subsequent anthropometric measurements (at 6, 24, and 59 months of age), we used a spring scale with a maximum capacity of 24 kg. All children were weighed with the least amount of clothing possible and without shoes. During the length assessment, we used a portable infant anthropometer for children younger than 2 years and a stadiometer for older children.

### 2.3. Assessment of Total Hair Hg (HHg) Concentration

Hair samples were collected from the occipital regions of mothers and children with stainless steel scissors. HHg levels were determined in samples obtained at the time of delivery and after 6, 24, and 59 months according to routine laboratory methods [14]. Briefly, hair samples were washed with a 0.01% ethylenediaminetetraacetic acid solution, rinsed with ultrapure water, and dried at 50 °C. The samples were then fractionated with stainless steel scissors to ensure better homogenization and increased efficiency during acidic digestion. After registering their weights, samples were digested with 5 mL of HNO_3_ and H_2_SO_4_ (1:1), as well as 4 mL of 5% KMnO_4_ in a digester block at 80 °C for 40 min. The analysis of HHg was performed by atomic absorption spectrophotometry with cold vapor generation in a Perkin-Elmer^®^ FIMS-400 flow-injection system (Ueberlingen, Germany). The results were expressed in μg/g of total Hg [14].

### 2.4. Assessment of Anemia

The anemia assessment (at 24 and 59 months) was conducted with a HemoCue^®^ portable photometer (hemoglobinometer, Angelhoim, Sweden). Blood samples from a digital puncture were collected in disposable microcuvettes and hemoglobin concentrations determined according to the manufacturer’s instructions. The results were obtained immediately and expressed in grams per deciliter (g/dL). The instrument was calibrated according to the manufacturer’s specifications. Children were considered anemic if their hemoglobin levels were below 11 g/dL [19].

### 2.5. Study Variables and Statistical Analysis

The influence of maternal fish consumption on the anthropometric indices of the children was assessed through the changes in the mean Z-score of the H/A, W/A, and W/H parameters during the study period. The anthropometric indices (W/A, H/A, and W/H) were calculated using the WHO Anthro version 3.2.2 [20]. The WHO growth curves for children from birth to 5 years of age were used as references. Z-score values below −2 and above +2 were used as cut-off values to define weight-stature deficits and excess weight, respectively. 

The data were analyzed using a linear mixed-effect model. This model allows for the analysis of repeated and correlated measures of the same child over time. To model the expected value of the response variable (Z-score of W/A, H/A, and W/H), the following variables were considered as fixed effects: living location, weight, length, gestational age, sex, maternal age, parity, HHg levels in the newborn, maternal HHg levels, family income, maternal education in years, frequency of fish consumption, breastfeeding, age of the child, and hemoglobin levels. The random effects (subject and time) were used to model their covariance structure [21].

Collinearity among the independent variables was tested using the Pearson correlation test. Variables with an r coefficient >0.80 were further tested for multicollinearity through the variance inflation factor (VIF) test. A VIF cut-off of <2.5 was used to determine which variables would remain in the model. After this step, the variables length at birth and total HHg level of the study child were excluded.

The next step began with the construction of a reduced model for the W/A, H/A, and W/H parameters along with the variables sex, age, and maternal HHg. We assessed the behavior of these variables as we introduced other independent variables, with variables having a significance level lower than 20.0% (*p* < 0.20) were eligible for further analysis. We used the backward strategy for the construction of the models for the W/A, H/A, and W/H parameters. The variables with a *p*-value <0.05 were maintained in the models. 

In order to select the best model for the W/A, H/A, and W/H parameters, we assessed candidate models using the Akaike’s Information Criterion (AIC) values based on the logarithmic similarity function between the adjusted models. This is calculated as AIC = −2L $\left(\hat{\theta }\right)$ + 2*d*; where L is the maximized similarity of the model and *d* represents the total number of parameters with fixed and random effects estimated by the model. The model with the lowest AIC value was considered the optimal model [22].

After variable adjustments, we conducted tests to verify whether the basic assumptions of a distribution for the mixed-effects models were met [21,22]. The behavior of the residuals was observed by comparing adjusted values to the residuals, and the theoretical quantiles of the standardized normal distribution to the quantiles observed in the sample. A histogram of the residues was used to verify normality and homogeneity.

To test the effect of the amount of fish consumed on maternal mercury levels according to the area of residence (urban and non-urban), we used the two-way analysis of variance, where the maternal mercury level had been subjected to Box-Cox transformation. Subsequently, the Tukey test was applied for multiple comparisons. The Shapiro-Wilk test was used to verify the normality of the data on mercury levels in children over time (0, 6, 24, and 59 months) and the periods were compared using the Friedman test. The level of significance was 0.05.

The height and weight curves were constructed using estimation methods for maximum similarity and maximum restricted similarity, respectively. The random effects for the intercept and the linear term for age (slope) were included to explain the intra-subject correlation measures in the assessment of variance estimates. The specification of the correlation structure was conducted using a first-order autoregressive matrix to describe correlations over time. The adjusted curves were smoothed by cubic splines for weight and height [22] and graphically compared with the WHO reference curves according to sex [23]. The statistical analyses were performed using R statistical software (version 3.3.2; R Development Core Team, Vienna, Austria).

## 3. Results

This study included 1433 women and their respective children, who were followed-up from birth until 5 years of age. The data losses throughout the study were only 4.2% and occurred in a randomized manner, with no significant effects on the analysis. The final sample (1373) consisted of 49.7% boys and 50.3% girls. Of those, 48% lived in urban areas and 52% lived in non-urban areas (riverside, rural area, and tin-mining settlements). Seventy-eight percent of children (both sexes) were born after full-term pregnancies with adequate mean weight and length. Only 1.9% of the children were underweight (<2500 g) at birth. During the follow-up period, the W/A, H/A, and W/H indices presented Z-scores between ≥−2 and ≤1 in almost 80% of the study population. Subjects with W/A, H/A, and W/H deficits occurred between 6 and 24 months, whereas being overweight for a given child’s height was more frequent at 24 and 59 months. Detailed characteristics of the subjects are described in Table 1.

Mothers were predominantly young, had low income and limited schooling (Table 1), with a mean breastfeeding duration of 5.6 months (urban) and 7.4 months (non-urban). The rate of teenage pregnancy (13–19 years) was notably high (*n* = 469; 34%); there were 65 mothers with age between 13–15 years (4.7%). However, the mean Z-scores, H/A (−0.02), W/A (−0.33), and W/A (−0.52) for these mothers’ children did not differ from those for the adult mothers’ children. 

**Figure 1:**
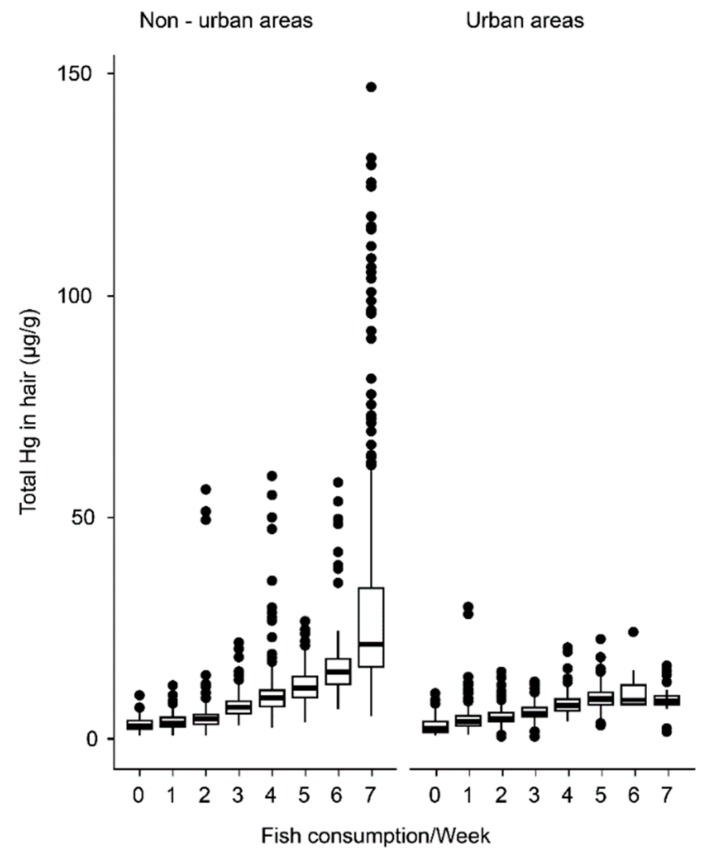
The relationship between maternal Hg levels and frequency of fish consumption in urban and non-urban areas. Box plot charts comparing the weekly fish meals with the total hair-Hg concentrations of urban and non-urban mothers (Tukey test: *p* < 0.0001).

The average maternal fish consumption, as measured by HHg levels, was higher over time in women from non-urban areas, as compared to those from urban areas (μ_d_ = 0.0687; *p* < 0.0001). In non-urban areas, HHg levels in mothers who consumed fish more than 3 times a week were above the limits recommended by the WHO (6 μg/g) (Figure 1).

**Figure 2:**
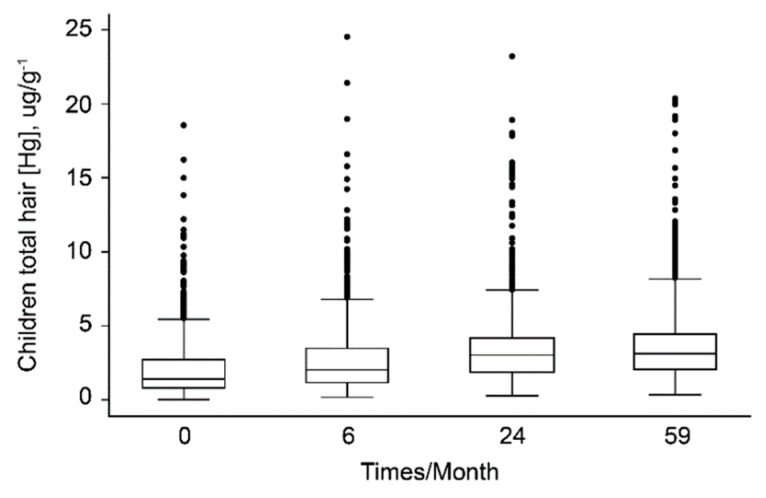
Hg levels in the hair samples of children over time (Friedman’s χ^2^ = 2270.3; *p* < 0.0001).

Based on hemoglobin concentrations, 618 (45%) children were considered anemic (hemoglobin levels <11 g/dL), of these 380 (27.7%) had mild anemia (hemoglobin levels 100–109 g/dL) and 238 (17.3%) had moderate anemia (hemoglobin levels 70–99 g/dL); there were no cases of severe anemia (hemoglobin levels <70 g/dL). However, higher hemoglobin levels and maternal age significantly affected the increase in the 3 anthropometric indices. All children in the study presented substantial increases in HHg levels over time (Friedman’s χ^2^ = 2270.3; *p* < 0.0001) (Figure 2). 

Our findings indicate a positive influence of higher family income on H/A (β = 0.0001, *p* < 0.0001) and W/A (β = 0.0008, *p* < 0.005) indices; however, W/H gain was associated with higher maternal education (β = 0.0181, *p* < 0.0001) (Table 2). Children whose mothers consumed fish more than 3 times a week had an overweight and obesity rate of 3.4%, as well as a height deficit of 2.7%. Weight at birth contributed positively to the increase in the H/A (β = 0.0006, *p* < 0.000) and W/A indices (β = 0.0008, *p* < 0.000) (Table 2). Maternal fish consumption in the presence of other variables did not remain significant after adjustments in the final models for the W/A, H/A, and W/H indices. 

A higher number of children were associated with low H/A rates (β = −0.0223, *p* = 0.03). Children born after a full-term pregnancy were taller (β = 0.0173, *p* = 0.03). The increase in W/H was positively influenced by breastfeeding time (β = 0.0053, *p* < 0.05). The H/A index decreased with increasing age (*p* < 0.0000). Boys presented with lower height (β = −0.4563, *p* < 0.0001) and weight gain (β = −0.4076, *p* < 0.0001) than girls. Children living in urban areas had lower W/H indices (β = −0.0743, *p* < 0.05) than non-urban areas (Table 2).

**Figure 3:**
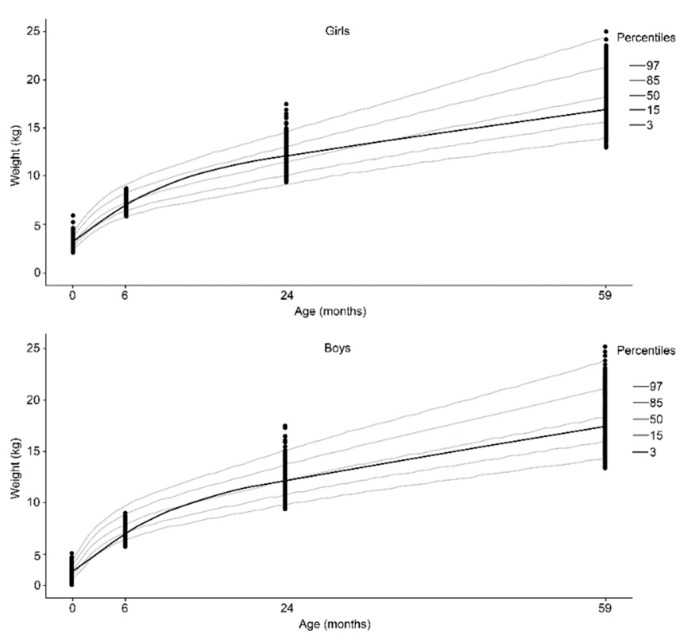
Weight according to age adjusted by the cubic spline function for both female (**upper**) and male (**lower**) sex.

**Figure 4:**
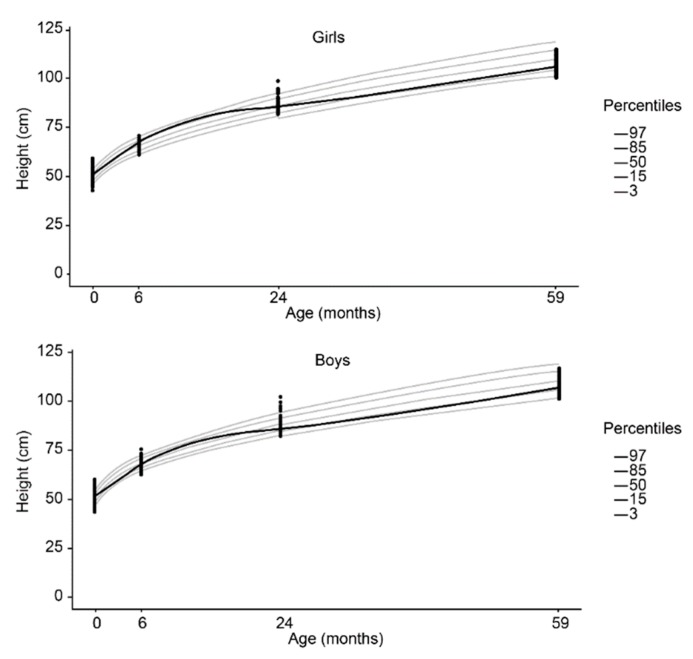
Height according to age adjusted by the cubic spline function for both female (**upper**) and male (**lower**) sex.

Compared to the WHO reference curves, we observed that the H/A and W/A indices for most children, regardless of sex, were greater than the 3rd percentile and no more than the 97th percentile (Figure 3 and Figure 4). Over time, the increase in W/A measurements were higher than the increases in H/A. Girls presented higher H/A and W/A indices than boys in relation to the reference median (Figure 3 and Figure 4). At 24 and 59 months, the H/A curves for both sexes presented a decrease in relation to the birth median (Figure 4).

## 4. Discussion

This study is unique in addressing HHg as a marker of habitual freshwater-fish consumption and exposure to MeHg (a major fish contaminant); therefore, it examined both the nutritional impact of dietary fish intake and the potential harm of MeHg on the growth of children in the first 5 years of life. Our main finding was that maternal fish intake, reflected by HHg, had no direct influence on the W/A, H/A, or W/H indices of the study children. Even children living under relatively unfavorable conditions presented anthropometric indices within acceptable limits, as defined by the WHO growth curves.

In populations where fish are an important source of contaminants, MeHg may be an important biomarker of fish consumption, especially in women [24]. Previous studies have shown that total concentrations of Hg in hair in Amazonian populations were significantly correlated with self-reported fish intake [12,13,15,25,26]. Due to the low accuracy of the data obtained through the questionnaires [27] the HHg biomarker seemed to be a better indicator for assessing the influence of the ingestion of fish on the growth of Amazonian children. In traditional Amazonian living, the limited purchasing power associated with regional sociocultural characteristics make fish a more accessible protein source than beef and chicken [13]. Given the wide variety of species available in the Madeira River basin, Hg concentrations in fish muscle tissue can range from 0.01 to 6.06 μg/g, depending on size and feeding behavior [28].

In the studied population, especially in non-urban areas, people tend to consume fish more frequently (1 or more times per day); consequently, they presented higher concentrations of Hg in their hair (Figure 1). The high maternal fish consumption influences total Hg levels in newborns and breastfeeding infants [29], which explains the observed increase in these children’s HHg levels over time (Figure 2). The advantages of breastfeeding are well established [30] and seem to compensate for the adverse effects of Hg [12,31]. Breastfeeding was a positive factor that contributed to an increase in the W/H index (Table 2). In fact, breastfeeding practices of Amazonians were a positive factor that contributed to infant anthropometric indices [32,33].

The results addressing the effect of a fish-based maternal diet on birth weight of these children were reported in an independent publication and showed no effect of maternal fish-Hg consumption [16]. Nevertheless, in our sample, birth weight was a positive predictor of stature (H/A) and weight (W/A) gain. The implications of family Hg exposure resulting from fish consumption on young children’s anthropometric indices can be contradictory. While some authors reported negative [34] and positive [35] influence of Hg on childhood anthropometric indices, others have not inferred such a relationship in their reports [15,36,37]. Still, others have indicated that Hg has no influence on Z-scores of height and weight indices in children ≤6-years-old; however, an increase in Hg concentrations was associated with a higher body mass index (BMI) [38].

Besides nutritional factors, growth of a child also depends on the living conditions in which the child is exposed [18,39]. In Amazonian populations with high fish intake, children’s anthropometric indices (H/A, W/A, and W/H) are influenced more by variables associated with socioeconomic and maternal-infant factors, rather than with fish-Hg exposure. The maternal factors, such as the mother’s age, showed a positive association with child growth; increasing maternal age was related to higher anthropometric indices (H/A, W/A, and W/H). It is worth noting the high frequency of teenage pregnancy (34%) in this study; this pregnancy rate was higher than that observed in the State of Rondônia (20.1%) and Brazil as a whole (17.7%) [40].

For low-income families, living in urban areas in the Amazon does not favor a better anthropometric outcome than rural living; children from urban areas had a lower W/H gain. Our findings also indicated a positive influence associated with higher family income on the H/A and W/A indices. This confirms previous findings that associated unfavorable socioeconomic and environmental conditions with a high prevalence of anthropometric deficits in Amazonian children [18,39,41,42,43].

Indeed, poverty in the Amazon has been associated with growth delay in children [18,39,41,43] and is aggravated in indigenous children [44,45]. The risk of malnutrition increases in response to the transition from breastfeeding to often inadequate feeding. In addition, there is also an increased risk of disease due to greater interactions with unfavorable environments [1,17,46]. Dórea et al. [12] considered diseases, such as malaria, and precarious health services may be problems of greater magnitude for indigenous people in the Amazon than the consumption of fish contaminated with mercury.

However, researchers suggest that Amazonian children could have a different growth potential from other regions around the world; therefore, the use of specific references based on local populations would be more appropriate than those established by the WHO [47,48]. The increasing age of the children was accompanied by an increase in the W/H ratio. Although the prevalence of overweight has been low, the increase in W/H with age deserves attention in the Amazon region. In addition, studies in remote areas of the Western Amazon showed that the impact of economic development increased the prevalence of overweight children [49,50]. Although additional studies are needed, it is likely that children of certain Amazonian populations will have a greater increase in weight than in height as they age [48].

Furthermore, the growth of Amazonian children is marked by nutritional inadequacy. As children grow older there is a greater demand for nutrients. The standard diet of the Amazonian population is based mainly on fish and cassava flour (which has a low energy density). Therefore, it is possible that complementary feeding may be insufficient to meet the micronutrient needs of growing children [11]. This could partially explain the high frequency of anemia (45%), especially at 59 months, in our study population.

Overall, maternal fish intake (estimated as HHg at the end of pregnancy and during lactation) had no direct influence on the W/A, H/A, or W/H indices of Amazonian children. Due to concerns about the early exposure of children to Hg through maternal fish consumption, international agencies have recommended restricting its consumption to 3 servings per week [51]. We must consider that dietary fish intake opposes the possible risks of Hg exposure for Amazonian populations. In such unfavorable socioeconomic and environmental conditions, habitual fish consumption contributes to a balanced diet and contains a high biological protein value for pregnant women and children [13].

In the face of socio-economic limitations, which affect families residing in this region, and in addition to accompanying food insecurity, it is counterintuitive to encourage the substitution of fish for foods that they are not accustomed to consuming.

## 5. Limitations

Considering that our study was designed to evaluate environmental issues related to Hg exposure and child development, there are limitations pertaining to the lack of information regarding diet and nutrition of mothers and infant complementary feeding which are the important factors in determining growth. The lack of complete information on maternal diet, as well as other pertinent environmental information related to lifestyle constitutes an important limitation of this study. Due to logistical difficulties, it was not possible to perform random sampling, but for the chosen areas, we could enroll most of the families. However, the strength of this study lies in the monitoring of individuals over time in an area where field logistics are considerably more difficult to obtain and where most research involving children is conducted using cross-sectional studies. Therefore, our results on Hg exposure and fish consumption are reliable.

## 6. Conclusions

High consumption of freshwater fish by pregnant mothers did not affect the anthropometric indices of children followed-up from birth until 5 years of age. However, we recommend controlling the consumption of high-Hg fish species by pregnant women and children during early development.

## Figures and Tables

**Table 1:** Characteristics of the 1343 pairs of mothers and children comprising the 5-year Rondônia study population.

Urban					Non-Urban			
Variables	Min	Mean	Max	(±SD)	Min	Mean	Max	(±SD)
**Mothers**								
Income ^1^	100	752.0	4500	504.49	50	560.60	2500	338.95
Maternal schooling in years	0	6.80	16	3.28	0	5.22	16	2.73
Number of children	0	1.96	8	1.60	0	2.00	12	1.80
Maternal age (years)	13	23.05	42	6.09	13	22.60	43	5.87
Gestational age (week)	35	39.33	45	1.53	32	38.91	43	1.62
Fish consumption per week	0	2.27	7	1.44	0	3.30	7	2.32
Breastfeeding (months) ^2^	0	5.6	36	5.58	0	7.4	40	5.82
HHgM (µg/g) ^3^								
0 months	0.73	5.56	29.32	3.09	1.02	11.61	253	17.02
6 months	0.5	4.95	19.59	2.41	0.7	10.49	125.21	13.79
24 months	0.49	5.66	29.72	2.83	0.87	10.76	129.15	13.35
59 months	0.55	5.67	15.84	2.69	0.56	11.28	146.87	14.52
**Children**								
Length at birth (cm)	43	50.99	59.5	2.66	43	50.5	59	2.57
Weight at birth (g)	2200	3281	5950	498.03	2010	3150	5250	422.05
H/A (Z-score) ^4^								
0 months	−2.58	0.76	5.56	1.42	−3.14	0.65	2.29	1.35
6 months	−3.13	0.24	2.29	1.06	−3.13	0.22	2.48	1.06
24 months	−2.03	−0.32	4.86	0.92	−2.23	−0.31	3.20	0.80
59 months	−1.83	−0.71	1.23	0.57	−1.94	−0.69	1.02	0.57
W/A (Z-score) ^4^								
0 months	−2.67	−0.41	506	1.02	−2.53	−0.18	3.81	0.88
6 months	−2.92	−0.76	1.24	0.69	−2.84	−0.69	1.45	0.71
24 months	−2.25	0.12	3.19	0.93	−2.16	0.20	3.09	0.83
59 months	−2.42	−0.54	2.29	0.93	−2.42	−0.47	2.15	0.93
W/H (Z-score) ^4^								
0 months	−4.19	−1.03	2.75	0.88	−4.34	−1.08	2.38	0.93
6 months	−3.94	−1.11	2.43	0.83	−2.57	−1.01	1.5	0.70
24 months	−3.53	0.35	3.88	1.39	−4.11	0.44	4.78	1.23
59 months	−2.38	−0.21	3.48	1.01	−2.45	−0.13	3.05	0.95
HHgC (µg/g) ^3^								
0 months	0.0001	2.26	24.55	1.74	0.0001	3.53	19.99	2.70
6 months	0.0001	2.24	15.65	1.60	0.0001	3.41	23.24	2.72
24 months	0.0001	2.28	21.41	1.85	0.0001	3.49	18.53	2.56
59 months	0.0001	2.18	12.83	1.61	0.0001	3.42	16.58	2.34
Hemoglobin (g/dL)								
24 months	8.2	10.96	13.0	1.03	8.1	11.02	13.4	1.01
59 months	8.3	10.80	13.1	1.02	8.1	10.87	12.8	0.96

^1^ Local currency (Real); ^2^ sampled at the end of lactation; ^3^ HHgM, HHgC = Mercury in hair of mothers (M) and children (C), respectively; ^4^ H/A, height-to-age; W/A, weight-to-age; W/H, weight-to-height.

**Table 2:** Multiple analyses of the linear mixed-effect model with random intercept and slope for H/A, W/A, and W/H indices in children from Rondônia State, Brazil.

Fixed Effect	β	SE	95% CI	*p*-Value	Random Effect	Variance	SD	Model Adjustment	
**H/A**									
Intercept	−2.182	0.3081	−2.786, −1.578	<0.0001	Intercept	0.5757	0.7587	−2 Log-Similarity	−7604.6
Child age	−0.0200	0.0006	−0.0212, −0.0187	<0.0001	Child age	0.0779	0.2791	AIC ^2^	15,233.2
Male	−0.4563	0.0247	−0.5047, −0.4078	<0.0001	Residue	0.8323	0.9123		
Number of children	−0.0223	0.0105	−0.0429, −0.0017	0.0339					
Birth weight	0.0006	<0.0001	5.4 × 10^−4^, 6.5 × 10^−4^	<0.0001					
Income ^1^	0.0001	<0.0001	6.5 × 10^−5^, 1.8 × 10^−4^	<0.0001					
Gestational age (weeks)	0.0173	0.0080	0.0016, 0.0331	0.0312					
Hemoglobin ^3^	0.2288	0.0121	0.2306, 0.2775	0.0000					
Maternal age (years)	0.0081	0.0030	0.0022, 0.0140	0.0072					
Residential location	0.0075	0.0261	−0.0436, 0.0586	0.7736					
Maternal schooling (years)	−0.0016	0.0046	−0.0105, 0.0074	0.7312					
Breastfeeding	0.0041	0.0022	−0.0001, 0.0084	0.0579					
HHgM ^4^	0.0006	0.0015	−2.3662, 0.0037	0.6656					
Weekly fish intake	−0.0107	0.0064	−0.0232, 0.0017	0.0921					
**W/A**									
Intercept	−2.939	0.0982	−3.131, −2.746	<0.0001	Intercept	0.2744	0.5238	−2 Log-Similarity	−6983.1
					child age	0.1103	0.3321	AIC ^2^	13,986.2
Male	−0.4076	0.0236	−0.4538, −0.3613	<0.0001	Residue	0.5776	0.7600		
Birth weight	0.0008	<0.0001	7.8 × 10^−4^, 8.9 × 10^−4^	<0.0001					
Income ^1^	<0.0001	<0.0001	2.8 × 10^−5^, 1.3 × 10^−4^	0.0027					
Maternal age	0.0048	0.0020	0.0009, 0.0087	0.0147					
Hemoglobin ^3^	0.2442	0.0183	0.2880, 0.3584	0.0000					
Maternal age (years)	0.0078	0.0077	−0.0073, 0.0229	0.3122					
Residence location	−0.0298	0.0246	−0.0780, 0.0185	0.2272					
Maternal schooling (years)	0.0054	0.0044	−0.0031, 0.0141	0.2137					
Breastfeeding	0.0032	0.0020	−0.0008, 0.0072	0.1227					
Number of children	−0.0182	0.0100	−0.0379, 0.0146	0.0697					
Gestational age (weeks)	0.0078	0.0077	−0.0073, 0.0229	0.3122					
HHgM ^4^	0.0006	0.0015	−0.0023, 0.0036	0.6824					
Weekly fish intake	−0.0043	0.0063	−0.0167, 0.0081	0.4958					
**W/H**									
Intercept	−1.050	0.0689	−1.185, −0.915	<0.0001	Intercept	<0.000	<0.000	−2 Log-Similarity	−8474.0
Age	0.0162	0.0006	0.0150, 0.0174	<0.0001	Child age	<0.000	<0.000	AIC	16,970.0
Male	−0.0743	0.0304	−0.1339, −0.0147	0.0145	Residue	1.264	1.124		
Urban area	−0.0743	0.0319	−0.1368, −0.0118	0.0198					
Breastfeeding	0.0053	0.0027	7.1 × 10^−5^, 0.0105	0.0470					
Maternal age (years)	0.0062	0.0025	0.0012, 0.0112	0.0151					
Maternal Schooling (years)	0.0181	0.0051	0.0081, 0.0281	0.0004					
Hemoglobin ^3^	0.2955	0.0220	0.3159, 0.3984	0.0000					
Income ^1^	<0.0001	<0.0001	−5.31 × 10^−6^, 0.0001	0.0681					
Number of children	−0.0188	0.0130	−0.0445, 0.0063	0.1403					
Gestational age (weeks)	−0.0058	0.0101	−0.0255, 0.0140	0.5669					
Birth weight	<0.0001	<0.0001	−2.81 × 10^−5^, 0.0001	0.2661					
HHgM ^4^	−0.0014	0.0015	−0.0044, 0.0017	0.3765					
Weekly fish intake	0.0151	0.0099	−0.0043, 0.0345	0.1280					

^1^ Local currency (Real); ^2^ AIC, Akaike information criterion; ^3^ Multiple analyses of the linear mixed-effect models at 24 and 59 months; ^4^ HHgM, mercury levels in the hair of mothers; SD, standard deviation; SE, standard error; 95% CI, 95% confidence interval.
